# Atypical primary pulmonary meningioma: a report of a case suspected of being a lung metastasis

**DOI:** 10.3332/ecancer.2014.414

**Published:** 2014-03-31

**Authors:** Daniela Lepanto, Fausto Maffini, Francesco Petrella, Marzia Colandrea, Carlo Putzu, Massimo Barberis, Giovanni Paganelli, Giuseppe Viale

**Affiliations:** 1 Division of Pathology, European Institute of Oncology, 20141 Milan, Italy; 2 Division of Thoracic Surgery, European Institute of Oncology, 20141 Milan, Italy; 3 Division of Nuclear Medicine, European Institute of Oncology, 20141 Milan, Italy; 4 Division of Medical Oncology, University of Sassari, 07100 Sassari, Italy; 5 University of Milan School of Medicine, 20122 Milan, Italy

**Keywords:** atypical meningioma, lung metastasis, meningioma

## Abstract

Primary extracranial and extraspinal meningiomas are very rare tumours, and primary pulmonary ones are even more uncommon. They present as a solitary pulmonary nodule, and most of them are benign, except for three cases. We describe a primitive atypical pulmonary meningioma first suspected of being a metastasis in a patient during follow-up ten years after therapy for breast cancer.

## Introduction

Extracranial and extraspinal meningiomas are very rare tumours that usually occur in the regions of the head and neck. Few cases have been reported in the lung, presenting as a solitary nodule. Most of these lesions are benign except for three cases [1–4]. Different histogenetic and pathologic mechanisms have been proposed, but the true aetiology of this tumour is still uncertain.

We present a new case of atypical primary pulmonary meningioma thought to be a solitary lung metastasis.

## Case report

A 60-year-old woman presented with a solitary pulmonary nodule during follow-up for a left breast infiltrating mixed ductal–lobular carcinoma, followed by CT + RT (chemotherapy plus radiotherapy 60 Gy), ten years earlier. It was thought to be a metastasis after ten years, so the nodule was detected in a restaging for breast cancer.

On CT evaluation, a single nodule was detected in the superior lobe of the left lung, 1.7 cm in diameter, suspected of being a metastasis.

**Figure 1.**
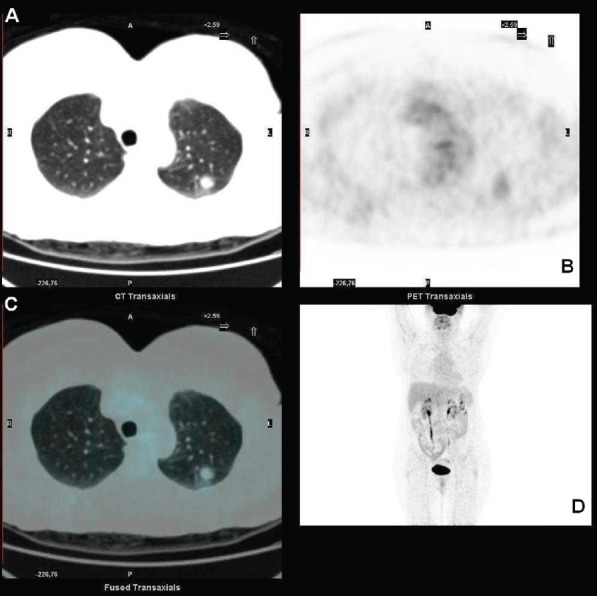
FDG-PET showing the pulmonary lesion in the upper lobe (a) transaxial low dose CT image, with midly increased metabolism (b) transaxial PET image, fused transaxial section and maximum intensity projection image (c and d).

Standard F18-fluorodeoxyglucose (18F-FDG) computed tomography–positron emission tomography (CT–PET) scan was performed and showed a mildly metabolitically active lesion suspicious for hamartoma or pulmonary metastases. At this site, the maximal standardised uptake value corrected for body weight (SUV bw_max) was 1.2 (Figures 1a–d).

No other extrapulmonary sites of increased FDG uptake were detected.

Perfusional pulmonary scintigraphy with a quantitative study of lung function was performed for hypothetical lobectomy and showed a non-specific bilateral reduction of pulmonary perfusion.

A single opacity was detected in the superior lobe of the left lung on the chest x-ray.

Bronchoscopy with transbronchial biopsy was performed and histology revealed chronic inflammation and extravasation of blood in pulmonary tissue: not diagnostic for malignancy.

Cytologic examination revealed an absence of tumour cells.

This was followed by a transthoracic fine needle biopsy with a diagnosis of a proliferation of mesenchymal and spindle cells. Immunohistochemical stains revealed positivity for epithelial membrane antigen (EMA) and progesterone receptors; negativity for AE1/AE3 cytokeratines, HMB45, caldesmon, estrogen receptors, TTF-1, p63, and HER-2. The final histologic diagnosis was heterotopic meningeal proliferation of lung.

A wedge resection of the nodule was performed with intra-operative histological examination that described a mesenchymal proliferation, negative for malignancy.

The tumour was a well-demarcated nodule, 1.6 cm in diameter.

**Figure 2.**
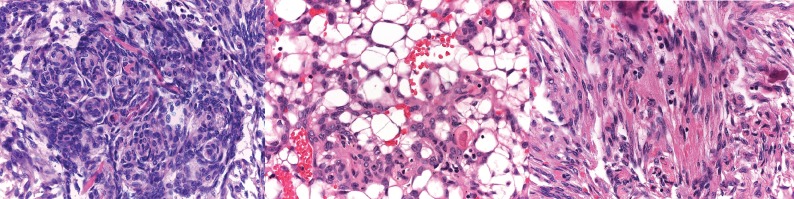
(a) Meningotheliod feature constituted by whorl of spindle cells. (H&E 20×). (b) Microcystic feature of small cells, with a clear cytoplasm (H&E 20×). (c) Atypical mitosis in a spindle cell area of meningioma typically observed in atypical meningioma of CNS (H&E 40×).

Microscopically, it was composed of bundles of mesenchymal and spindle cells, often arranged in whorls, with fibroblastic, meningeal, and microcystic features (Figures 2a and b). Atypical mitotic figures (4/10 HPF) were present (Figure 2c). Immunohistochemical stains revealed positivity for EMA and progesterone receptors and negativity for AE1/AE3 cytokeratins and inhibin.

The final histological diagnosis was atypical meningioma of the lung.

The central nervous system RMN was performed in another hospital and was reported to be negative. After one year of follow-up the patient is still alive without evidence of metastasis or recurrences.

## Discussion

Primary pulmonary meningioma is a very rare tumour. Since the first case was described by Erlandson in 1981, about 40 cases (including our report) have been reported in the medical literature, to the best of our knowledge [1–4]. Only three of these cases showed malignant characteristics as highest mitotic activity, necrosis, lymph-node or distant metastasis.

Meningiomas of central nervous system grow slowly without early clinical symptoms. Later, patients show a compressive sign of neoplasm as headache or sensitive-motor deficit; in contrast, lung lesions, although they present most often as an asymptomatic solitary pulmonary nodule, rarely show signs of bronchial stenosis followed by bronco-pneumonia.

In general, the nodule is detected in staging during follow-up for a previous cancer or tuberculosis, or during routine screening studies or incidentally [4].

Diagnosis is made by histopathological analysis after the accidental demonstration of enhancement on CT scan and uptake at FDG-PET or receptor imaging with somatostatin analogues, although primary pulmonary meningioma are not normally studied by CT–PET scanning, in the setting of a known malignancy and routine oncologic follow-up, because of a false-positive diagnosis of metastasis [5]. Therefore, the diagnosis should be taken into consideration of FDG avid masses of the lung during oncologic follow-up. When imaging results are not conclusive, transthoracic needle biopsy and surgical resection are mandatory [3].

The histologic differential diagnosis includes primary and metastatic spindle and clear cell tumours of the lung, pulmonary metastasis, and solitary fibrous tumour of the pleura; immunohistochemical assay can safely identify this kind of neoplasm [6].

Benign tumours are generally well circumscribed, with a median size of 1.8 cm; the only three malignant meningiomas are 5, 6.5, and 15 cm in diameter. Our case of meningioma of the lung was 1.6 cm in diameter but with atypical histological features. Meningioma of CNS, following a WHO grading system [7], are classified into three grade of malignancy: benign, atypical, and malignant. In the literature, there are data concerning the prognosis of extracranial–extraspinal both benign and malignant meningiomas but not of the atypical ones. The mitotic count and the necrosis are the most important features that should be evaluated when grading meningiomas. A radiological study of the CNS, preferably an MRI, is required to exclude an intracranial or spinal meningioma. In fact, meningiomas of the central nervous system arise in the cranial cavity and spinal cord and rarely metastasise outside the skull or spinal cord, although more than 80 cases of metastatic meningiomas of the lung have been reported in [3].

The pathogenesis is uncertain: it may arise 35 years after low-dose exposition radiotherapy (RT) and 19–24 years after high-dose RT. The post-RT meningioma is more atypical than others [7–9]. The atypical meningioma we have described arose ten years after RT, at the same thoracic side of a previous breast carcinoma. Therefore, it could be a radio-induced meningioma and this could explain the atypical features [7–9]. Same authors explain the origin of lung meningioma from pluripotential sub-pleural mesenchyma or from heterotopic embryonic rests of arachnoid cells as pulmonary meningothelial nodules that show a similar immunohistochemical pattern [10]; however, a genotypic comparison supports a different histogenesis [3, 6].

The role of estrogen and progesterone receptors in this kind of tumour is still unclear and does not explain the difference observed between genders [6, 7]: in fact, women are more affected than men.

The extracranial meningioma and, in particular, the lung one should be treated by surgery, as first-choice therapy. Intra-operative frozen sections are very important to prove free margins. If the resection is complete, there is no risk of recurrence for benign forms, while the prognosis is still unclear for atypical ones because of the absence of data. The intra-operative frozen section can also prevent an over-treatment [3]. After one year of follow-up following a wedge resection, the patient is still alive without evidence of metastasis or recurrences.

## Conclusion

In conclusion, pulmonary primitive meningioma is a very rare lesion, usually incidentally detected and generally benign, that can mimic other pulmonary tumours, such as metastasis. Resection is required for both diagnosis and treatment and free margins are necessary to avoid a recurrence for benign cases.

## References

[1] Van der Meij JJC (2005). Primary pulmonary malignant meningioma. Ann Thorac Surg.

[2] Prayson RA, Farver CF (1999). Primary pulmonary malignant meningioma. Am J Surgical Pathol.

[3] Incarbone M (2008). Primary pulmonary meningioma: report of a case and review of the literature. Lung Cancer.

[4] Weber C (2013). Primary pulmonary malignant meningioma with lymph node and liver metastasis in a centenary woman, an autopsy case. Virchows Arch.

[5] Picquet J (2005). Primary pulmonary meningioma first suspected of being a lung metastasis. Ann Thorac Surg.

[6] Drlicek M (1991). Pulmonary meningioma: immunohistochemical and ultrastructural features. Am J Surg Pathol.

[7] Perry A (2007). Meningeal Tumours WHO Classification of Tumours of the Central Nervous System.

[8] Michael J (1991). Radiation-induced meningiomas: experience at the Mount Sinai Hospital and review of the literature. J Neurosurg.

[9] Musa BS, Pople IK, Cummins BH (1995). Intracranial meningiomas following irradiation--a growing problem. Br J Neurosurg.

[10] Spinelli M (2000). Primary pulmonary meningioma may arise from meningothelial-like nodules. Adv Clin Pathol.

